# Sclerosing Encapsulating Peritonitis Mimicking an Internal Hernia: A Case Report

**DOI:** 10.7759/cureus.28476

**Published:** 2022-08-27

**Authors:** Mohamad Mansour, Yousef S Alabrach, Mahmoud Eladl, Khalid E Attia, Ibrahim El Nogoomi

**Affiliations:** 1 Clinical Sciences Department, College of Medicine, University of Sharjah, Sharjah, ARE; 2 Department of Radiology, Kuwait Hospital, Sharjah, ARE; 3 Department of General Surgery, Kuwait Hospital, Sharjah, ARE

**Keywords:** diagnostic laparoscopy, small bowel obstruction, acute abdomen, internal hernia, sclerosing encapsulating peritonitis, abdominal cocoon

## Abstract

Sclerosing encapsulating peritonitis (SEP) is a rare entity that could lead to abdominal obstruction; however, despite being reported in several case series, its underlying pathophysiology is still unclear. A large proportion of SEP cases are diagnosed incidentally or after surgical exploration, which poses a great challenge to pre-operative diagnosis. We hereby report a case of a 33-year-old male patient who presented with cachexia and a clinical picture of complete small bowel obstruction. CT scan of the abdomen raised suspicion of an internal hernia, prompting explorative surgical evaluation. Laparoscopy showed encasement of the small bowel loops in a thick fibrocollagenous membrane characteristic of SEP. Laparotomy with adhesiolysis and membrane excision successfully led to the resolution of obstruction. Retrospective interpretation of the initial CT scan confirmed the presence of SEP’s characteristic radiological signs and provided an insight into how it contrasts with an internal hernia. This case provides an opportunity to highlight the differences between the two clinical entities and the pre-operative diagnostic strategies.

## Introduction

Sclerosing encapsulating peritonitis (SEP), classically termed abdominal cocoon, is characterized by chronic subclinical inflammation leading to the formation of a fibrocollagenous membrane encapsulating primarily the small intestine, possibly involving other abdominal contents [1]. The majority of cases occur in the presence of a predisposing condition, such as peritoneal dialysis and abdominal tuberculosis, falling under the term secondary SEP [2]. However, in idiopathic cases where a predisposing condition cannot be identified, it is termed primary SEP [3].

Early diagnosis of SEP has been a great challenge to surgeons, with most cases being diagnosed late or incidentally on the operative table during laparoscopy or laparotomy [3]. The management of SEP requires extensive adhesiolysis and careful membrane excision; thus, earlier diagnosis is imperative for better surgical planning and earlier intervention [1,3]. We hereby present a case of complete small bowel obstruction suspected to be due to an internal hernia, prompting surgical evaluation and revealing SEP of the small bowel. This case provides an opportunity to contrast the differences between the two clinical entities and their pre-operative diagnosis.

## Case presentation

A 33-year-old man presented to the emergency department with a history of colicky abdominal pain, nausea, vomiting, abdominal distension, and obstipation for two days. He had a similar episode 15 months prior to this presentation and was managed conservatively as a case of partial small bowel obstruction. His past medical history was unremarkable, with no known chronic illnesses and no prior surgeries. On examination, the patient was vitally stable. His general appearance was significant for cachexia, with a weight of 47 kg and a BMI of 16.45. A firm, tender, tubular-like abdominal mass measuring 15 x 6 cm was palpated, extending from the suprapubic region to the epigastrium situated slightly to the right of the umbilicus. He had no hepatomegaly or splenomegaly. Per-rectal examination revealed palpable intestinal loops on the anterior aspect of the lower rectum, with no feces or blood. The clinical picture was suggestive of acute intestinal obstruction.

A complete blood count with differential was normal apart from a hemoglobin level of 17.20 mg/dL, likely due to hemoconcentration. Biochemistry was significant for a total creatine kinase level of 2,606 IU/L. The liver and kidney functions, amylase, and electrolytes were all normal. Viral hepatitis and HIV serology were negative. Purified protein derivative testing was negative.

**Figure 1:**
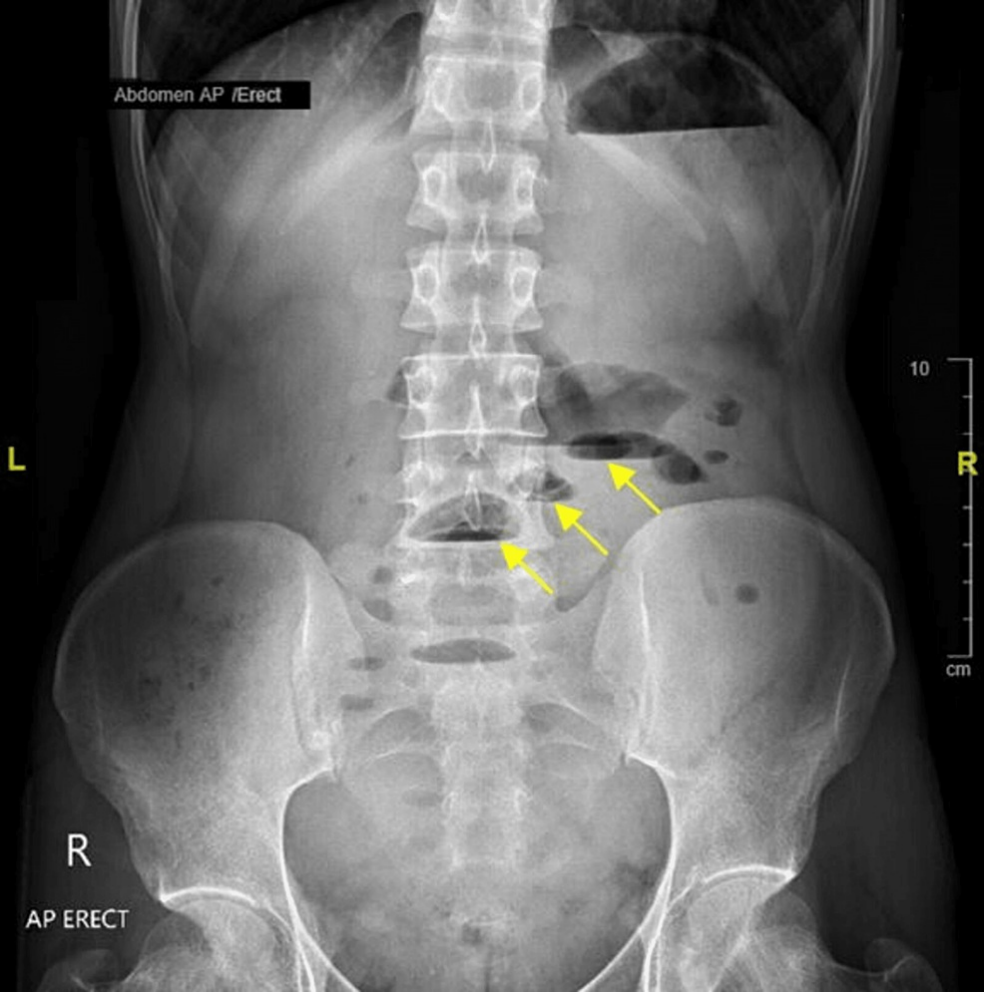
Erect abdominal X-ray at admission showing multiple air-fluid levels (yellow arrows), consistent with the clinical picture of acute small bowel obstruction.

**Figure 2:**
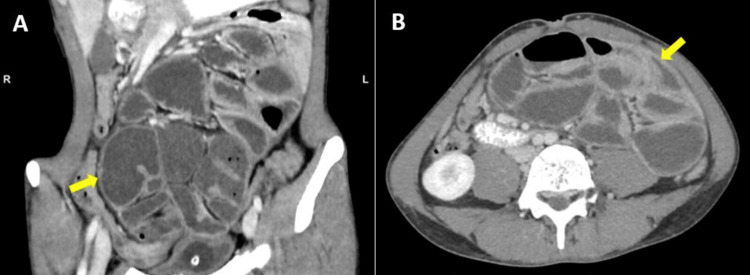
Coronal reconstruction (A) and axial slice (B) of abdominal CT with contrast showing clumped and dilated small bowel loops surrounded by an enhancing membrane (yellow arrows).

Erect abdominal X-ray showed multiple air-fluid levels (Figure 1). Abdominal CT with contrast (Figures 2A, 2B) revealed dilated small bowel loops with a convergent stretched mesenteric origin, along with collapsed large bowel loops distally, conforming with a small bowel obstruction due to internal hernia. It is noteworthy that the small bowel loops were surrounded by an enhancing membrane. A preliminary diagnosis of internal hernia was made. However, the CT findings raised suspicion of a possible different etiology, and diagnostic laparoscopy was considered.

**Figure 3:**
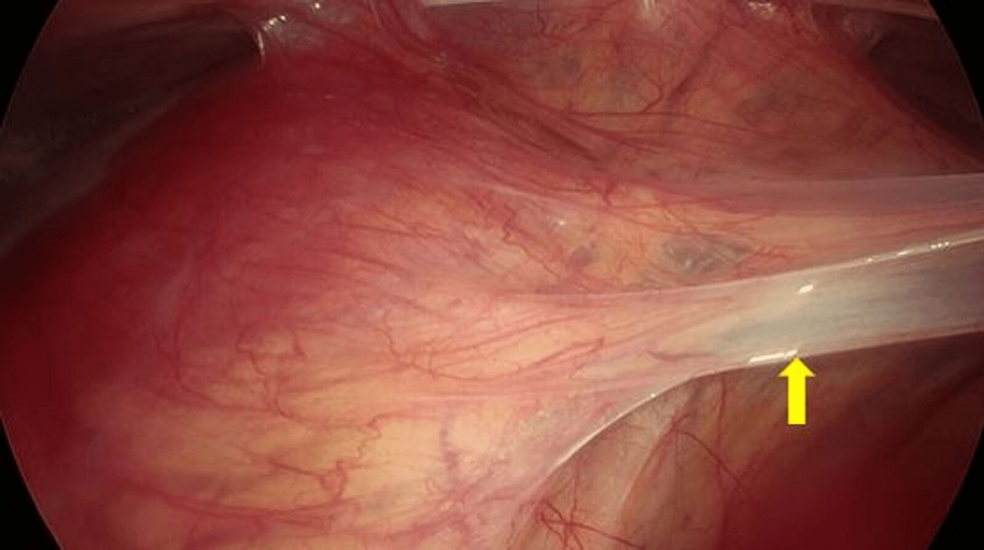
Laparoscopic view of the membrane with fibrous adhesions to the abdominal wall (yellow arrow).

**Figure 4:**
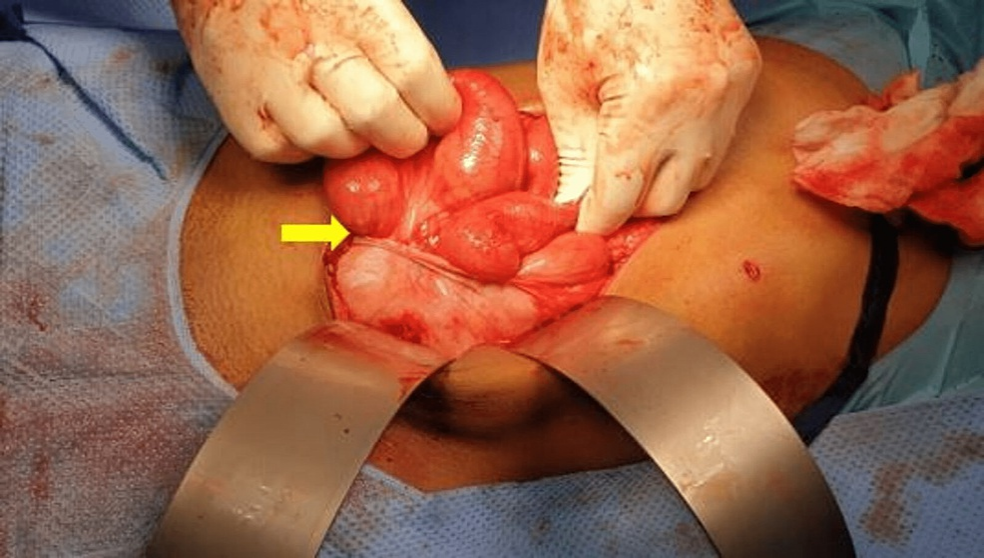
Appearance of the freed bowel loops after fibrous membrane resection (yellow arrow).

Diagnostic laparoscopy revealed a grey-white to tan fibrous membrane encapsulating the small bowel and part of the cecum, giving the classically described appearance of an “abdominal cocoon” (Figure 3). Calcifications were noted over the spleen. The rest of the organs appeared normal. Owing to the extensive adhesiolysis required, the laparoscopy was converted to laparotomy, where careful dissection of the fibrous membrane was performed, releasing the entrapped small bowel (Figure 4). Significant adhesions were found between the bowel loops, successful adhesiolysis was performed, and the freed intestinal segments were viable. The appendix was dissected from the adhesions, and an appendectomy was performed. Multiple biopsies of the fibrous membrane were taken. Placement of a pelvic drain was followed by closure of the laparotomy.

Histopathological examination of the membrane showed fragments of a fibrocollagenous wall of variable thickness with congested blood vessels and chronic inflammatory cell infiltrates. Sections studied from the appendix showed a complete loss of mucosa replaced by fibrosis, with few lymphoid follicles and no inflammation in the muscular or serosal layers.

The patient’s postoperative period was complicated by a left lower pelvic hematoma measuring 8 x 11 x 13 cm, which was successfully resolved after CT-guided drainage and empiric antibiotic therapy. Otherwise, the overall course was favorable with good recovery, and the patient was discharged on postoperative day 20. The patient was then lost to follow-up.

## Discussion

SEP has been classically described as an inflammatory condition of the peritoneum that results in encapsulation of the abdominal contents by a fibrous sac, potentially leading to bowel obstruction. It has been reported in different age groups ranging from adolescence to the elderly, with two to three times occurrence in males compared to females [1,4]. Previous studies have pointed to a greater predilection for populations of tropical and subtropical regions [3].

SEP has been classified according to anatomical involvement whether encasing the small bowel partially, totally, or also involving adjacent abdominal contents into type I, II, and III, respectively [4].

It is also further divided into primary (idiopathic) or secondary SEP [1]. Secondary SEP cases have been attributed to a multitude of etiologies such as peritoneal dialysis, abdominal tuberculosis, malignancy, and drugs [1,4]. A thorough history from the patient revealed none of these causes, and the CT findings, as well as the diagnostic laparoscopy, have shown involvement of the cecum and the spleen, rendering this case a primary type III SEP. Several case series have shown that type I and type II are more commonly encountered; however, it is not uncommon to find patients with type III, particularly with patients in advanced presentations such as our patient [1,4].

The preoperative diagnosis of SEP has proven to be challenging as seen in the majority of reported cases [1,3,4]. This can be justified by the vague and non-specific presentation occurring in episodes and reoccurring over several months to years [1]. Although the clinical picture may include abdominal pain, cachexia, loss of appetite, presence of an abdominal mass, and features of intestinal obstruction, a vast number of differentials can be attributed in these cases [4]. Consequently, most cases are diagnosed on the operative table [1]. As seen in our case, the presenting features of abdominal pain, cachexia, and visible lump, as well as the recurring episodes made early diagnosis challenging.

Thus, it is important to highlight the role of CT as a valuable pre-operative diagnostic tool for cases of suspected SEP, primarily in identifying and ruling out other differential diagnoses such as internal hernia, peritoneal encapsulation, or other causes of intestinal obstruction [3]. CT findings that are highly suggestive of SEP include clumping of bowel loops and presence of a thick enhancing membrane around the bowel loops. Additional CT features that are more specific in cases of acute obstruction include the cauliflower sign, concertina pattern, and bottle gourd sign [4]. As for our case, these signs were not easily identified pre-operatively, which led to the preliminary diagnosis of an internal hernia.

**Figure 5:**
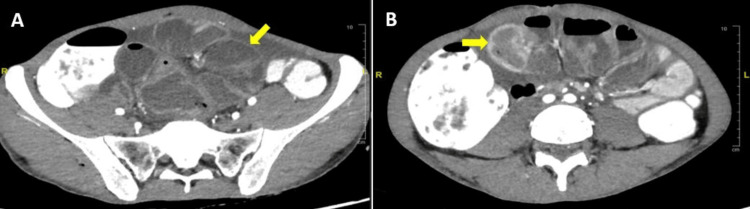
Axial slice of abdominal CT with contrast showing clumped small bowel loops in the center of the abdomen encapsulated by an enhancing membrane (yellow arrow) (A). Axial slice of abdominal CT with contrast showing a congregate of small bowel loops giving the appearance of an accordion or “concertina-like pattern” (yellow arrow) (B).

However, after the diagnosis was made intraoperatively and a thorough literature review was performed, the initial CT was reinterpreted and certain signs suggestive of SEP were appreciated. These signs included bowel clumping, presence of an enhancing membrane, and a concertina-like pattern. The CT images performed 15 months prior to the current presentation were also reviewed, and similar signs were seen (Figures 5A, 5B). These findings would have supported the diagnosis of SEP over internal hernia, thereby earlier surgical planning might have been feasible. Other important differentiating CT features between SEP and internal hernia are summarized in Table 1. In cases where CT findings are inconclusive, diagnostic laparoscopy is a valuable tool that can confirm the diagnosis and avoid potential complications during laparotomy [3].

**Table 1:** Differentiating CT features between SEP and internal hernia. *Main features. **Additional features SEP, sclerosing encapsulating peritonitis

Internal hernia	SEP
Centrally located cluster of dilated bowel loops in an abnormal anatomic location [2,5,6]	Congregated, dilated and adherent bowel loops in the center of the abdomen* [2,4,5]
No enhancing membrane is seen [2,5]	Enhancing membrane surrounding the bowel loops* [4,5]
Mass effect on adjacent organs [2,5]	Loculated or gross ascites** [2,4,5]
Displacement, stretching, engorgement, or crowding of mesenteric vessels [2,5]	Calcification over peritoneum, liver, or spleen** [2,4,5]
Evidence of small bowel obstruction [2,5]	Other characteristic signs (cauliflower, bottle gourd, concertina pattern, gingerbread man)** [2,4,5]

The management of SEP consists of excision of the fibrous membrane and subsequent adhesiolysis [2]. The majority of patients achieve resolution of obstruction in the immediate postoperative period [7]. However, the development of certain complications such as early postoperative small bowel obstruction, recurrent obstruction, wound site infections, intra-abdominal collections, and enterocutaneous fistulas can be attributable to several factors including acute presentation, delayed intraoperative diagnosis, extensive adhesiolysis, and bowel manipulation [1,7].

## Conclusions

SEP is a rare enigmatic entity that often presents with intermittent bowel obstruction. Due to its rarity, it is not usually included in the differential diagnosis and is therefore misdiagnosed. One of its differentials, internal hernia, can have overlapping clinical and radiological features. Understanding the subtle differences between the two entities can assist in pre-operative diagnosis, thereby contributing to better outcomes.
